# Profound and selective lymphopaenia in primary lymphatic anomaly patients demonstrates the significance of lymphatic-lymphocyte interactions

**DOI:** 10.3389/fimmu.2023.1279077

**Published:** 2023-11-03

**Authors:** Julian Pearce, Linda Hadcocks, Sahar Mansour, Malou van Zanten, Steve Jeffery, Kristiana Gordon, Pia Ostergaard, Peter Mortimer, Derek C. Macallan

**Affiliations:** ^1^ Lymphovascular Research Unit, Molecular and Clinical Sciences Research Institute, St George’s, University of London, London, United Kingdom; ^2^ Dermatology and Lymphovascular Medicine, St George’s University Hospitals NHS Foundation Trust, London, United Kingdom; ^3^ Institute for Infection and Immunity, St George’s, University of London, London, United Kingdom; ^4^ South West Thames Regional Centre for Genomics, St George’s University Hospitals NHS Foundation Trust, London, United Kingdom; ^5^ Infection and Immunity Clinical Academic Group, St George’s University Hospitals NHS Foundation Trust, London, United Kingdom

**Keywords:** lymphatic diseases, lymphoedema, lymphopaenia, cellulitis, lymphangiectasia, lymphocyte subsets, naïve T cells, WILD syndrome

## Abstract

**Introduction:**

The lymphatic system has a pivotal role in immune homeostasis. To better understand this, we investigated the impact of Primary Lymphatic Anomalies (PLA) on lymphocyte numbers and phenotype.

**Methods:**

The study comprised (i) a retrospective cohort: 177 PLA subjects from the National Primary Lymphatic Anomaly Register with clinical and laboratory data, and (ii) a prospective cohort: 28 patients with PLA and 20 healthy controls. Patients were subdivided using established phenotypic diagnostic categories and grouped into simplex (localised tissue involvement only) and systemic (involvement of central lymphatics). Further grouping variables included genital involvement and the likelihood of co-existent intestinal lymphangiectasia. Haematology laboratory parameters were analysed in both cohorts. In the prospective cohort, prospective blood samples were analysed by flow cytometry for markers of proliferation, differentiation, activation, skin-homing, and for regulatory (CD4^+^Foxp3^+^) T cells (Treg).

**Results:**

In patients with PLA, lymphopaenia was frequent (22% of subjects), affected primarily the CD4^+^ T cell subset, and was more severe in subjects with systemic versus simplex patterns of disease (36% vs 9% for lymphopaenia; 70% vs 33% for CD4^+^ cells). B cells, NK cells and monocytes were better conserved (except in *GATA2* deficiency characterised by monocytopaenia). Genital oedema and likelihood of concomitant intestinal lymphangiectasia independently predicted CD4^+^ T cell depletion. Analysing CD4^+^ and CD8^+^ T cells by differentiation markers revealed disproportionate depletion of naïve cells, with a skewing towards a more differentiated effector profile. Systemic PLA conditions were associated with: increased expression of Ki67, indicative of recent cell division, in naïve CD4^+^, but not CD8^+^ T cells; increased levels of activation in CD4^+^, but not CD8^+^ T cells; and an increased proportion of Treg. Skin-homing marker (CCR10, CLA and CCR4) expression was reduced in some patients with simplex phenotypes.

**Discussion:**

Patients with PLA who have dysfunctional lymphatics have a selective reduction in circulating lymphocytes which preferentially depletes naïve CD4^+^ T cells. The presence of systemic disease, genital oedema, and intestinal lymphangiectasia independently predict CD4 lymphopaenia. The association of this depletion with immune activation and increased circulating Tregs suggests lymphatic-lymphocyte interactions and local inflammatory changes are pivotal in driving immunopathology.

## Introduction

The lymphatic system has a pivotal, if often neglected, role in immune function (1). The immune system is largely accommodated within the lymphatic system, and lymphatic vessels are the primary transport route by which bacteria, foreign antigens, particulate matter and cutaneous immunological cells reach regional lymph nodes and lymphoid structures. Anatomically-constrained interactions with resident B and T lymphocytes and other cells are then crucial for the generation of both innate and adaptive immune responses.

Both the immune system and the lymphatic system develop and function in concert. It therefore follows that lymphatic dysfunction, whether primary or secondary in origin, will likely result in dysfunctional immunity (1, 2). A dysfunctional lymphatic system usually manifests clinically as lymphoedema. As carriage of antigen-presenting cells via the lymphatic vessels is pivotal in the generation of immune responses, lymphoedematous tissue can be considered as being in a state of ‘local immunodeficiency’ (1). Infection in this setting is common and lymphoedema is arguably the most important risk factor for cellulitis (3). One-third of patients with chronic oedema develop cellulitis at some point; the more severe the oedema, the more likely cellulitis is to occur (4). Patients often experience severe infections requiring prolonged courses of oral and/or intravenous antibiotics (5); some require continuous prophylactic therapy to control infection. It is not just bacterial infections that are more common with lymphoedema; recurrent viral warts commonly infect swollen limbs and can be very challenging to treat. Interestingly, patients with primary lymphatic anomalies (PLA) who have *GATA2* deficiency or ‘Warts, Immunodeficiency and Lymphatic Dysplasia’ (WILD) syndrome often have viral warts on unaffected, non-swollen tissue (6, 7), suggesting that immune dysfunction may be generalised as well as localised in those conditions.

The link between lymphocyte biology and the complex biological processes (lymphangiogenesis, inflammation, fibrosis) in lymphoedema pathogenesis has been well established experimentally – as reviewed in (8). For example, murine models show that the balance between Th1, Th2, Th17, and regulatory T cell (Treg) activity is pivotal in the genesis of lymphoedema. The converse effect – the impact of lymphoedema on lymphocyte biology - is much less well defined, especially in humans, not least because clinical models are limited. PLA however do represent an appropriate model system through which to assess the impact of a dysfunctional lymphatic system on circulating immune cell numbers because, despite constituting a heterogeneous group of conditions, their shared characteristic is an underlying primary developmental abnormality in the structure and function of the lymphatic system.

Patients with PLA present with swelling at birth, at puberty, or later in adulthood. Peripheral tissues, particularly the lower limbs, are most commonly involved, but any body part can be affected, including the face and genitals. Internal lymphatic dysfunction within the thorax or abdomen can result in accumulation of fluid, which is sometimes chylous; manifestations include pericardial/pleural effusions, ascites, chylous reflux, pulmonary lymphangiectasia, intestinal lymphangiectasia and protein-losing enteropathy (9). Many PLA have a genetic basis, either from germline or post-zygotic mutations. Over the last decade, many causal genes have been identified – at least 14 to date (9). Key to the identification of novel gene associations has been the practice of rigorous phenotyping, including investigations such as lymphoscintigraphy. This has allowed the clinical classification of PLA in a logical and reproducible manner (9, 10). An important clinical distinction can be made between those with likely involvement of the internal/central lymphatics, here referred to as having ‘systemic’ disease, and those without known internal involvement, referred to as having ‘simplex’ disease.

Although there are no systematic studies of white blood cell abnormalities in patients with PLA, in clinical practice we have observed markedly reduced circulating lymphocyte counts in patients with PLA and documented profound CD4 lymphopaenia in the subset of PLA patients with WILD syndrome (7). Investigations in patients with intestinal lymphangiectasia, not uncommonly associated with PLA, show depletion of CD4^+^ T-lymphocytes from blood with a preferential loss of naïve versus memory cells and a skewing of cytokine responses in memory cells towards a less inflammatory profile (more IL-4; less IFN-γ/IL-2) (11). Indeed, intestinal lymphangiectasia may be associated with profound cytopaenias in addition to the well-recognised loss of soluble factors such as immunoglobulins (12, 13). Such depletion of naïve CD4^+^ T cells appears to be associated with increased levels of activation, albeit without major disruption of T cell lymphoproliferative responses (14). Whether similar abnormalities to those seen in intestinal lymphangiectasia are observed in other PLA patients remains unknown, but such abnormalities are likely to be important, as depletion of circulating T cells would compromise both local and generalised immune function.

In this study, we took PLA as a model system for lymphatic dysfunction to interrogate the role of the lymphatic system in immune homeostasis. We first investigated how different leukocyte subsets were depleted in PLA; we then explored how patterns of lymphocyte depletion concur and differ between defined clinical entities; finally, we performed multi-parameter flow cytometry to characterise circulating T cells in terms of their activation status and their proliferative, regulatory and skin-homing profile. Taken together, the results help elucidate the role of lymphatic function in immune physiology.

## Methods

### Patients and subjects

Subjects were recruited from two cohorts, one retrospective and one prospective.

#### Retrospective cohort

The retrospective cohort comprised 177 subjects (median age 29, range 3-77; 84F:93M) with primary lymphatic anomalies (PLA) from the National Primary Lymphatic Anomaly Register (held at St George’s University Hospitals NHS Foundation Trust, London) with documented haematology and immunology indices collected between 2006 and 2020 (**Table 1**). No controls were included but laboratory normative data were used to derive the proportion of results for any cell type or patient-group that fell below the ‘Lower Limit of Normal’ (%LLN), allowing comparisons of cell types with different reference ranges. Reference ranges were age-specific and changes over time were normalised as described (15).

**Table 1:** Frequency of cytopaenia by cell type and clinical syndrome in Primary Lymphatic Anomaly (PLA) subjects.

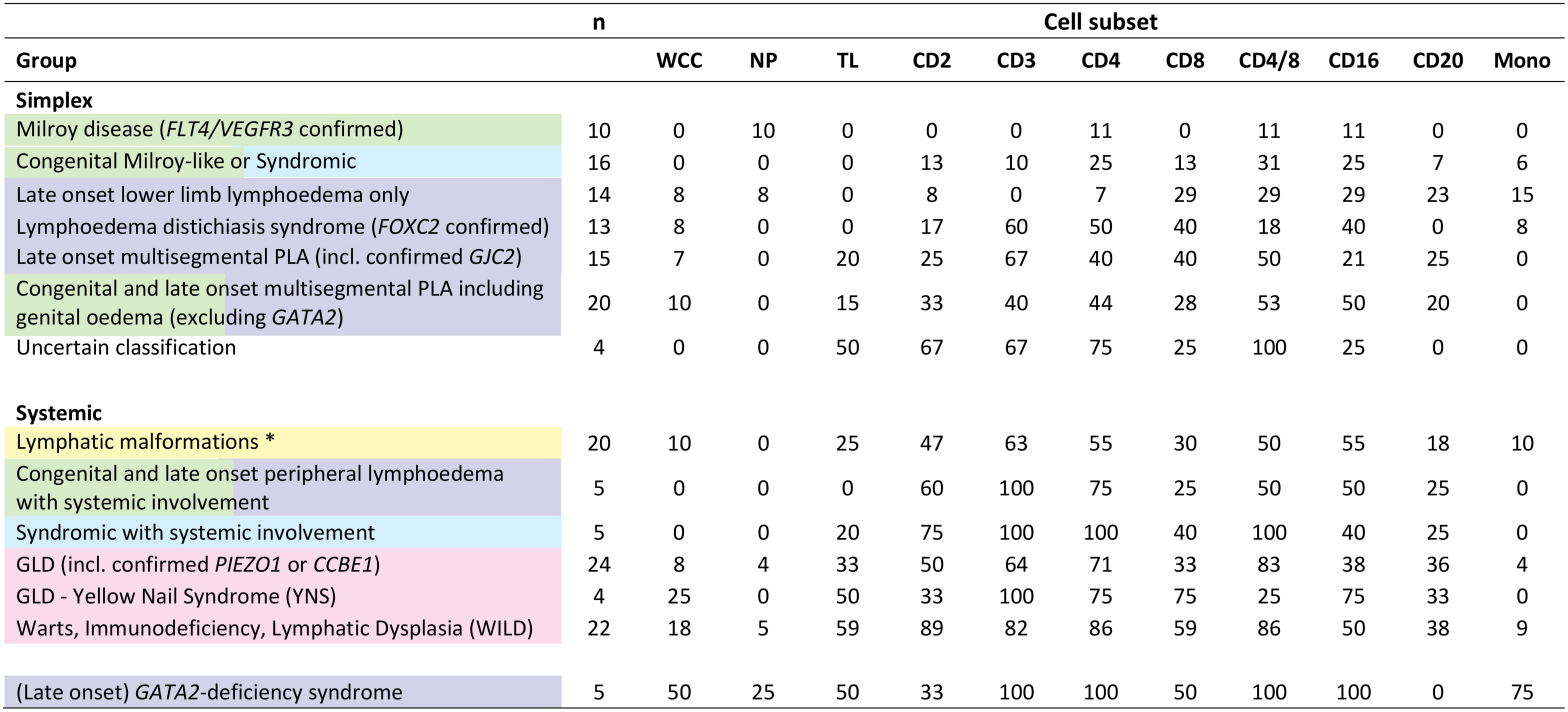

Data from the retrospective cohort of 177 subjects with primary lymphatic anomalies (PLA) from the National Primary Lymphatic Anomaly Register. Table shows subjects by diagnostic category grouped according to the St George’s clinical classification algorithm [**Figure S1**, (9)]; colours refer to the colour code in that classification; genotypes are given where known. Subjects with GATA2-deficiency syndrome are shown for comparison only. Data shows number (n) of subjects in each category and percentage of those subjects with a cell count below the lower limit of normal (%LLN) for each specific cell subset. * Lymphatic malformations described in **Table 1** include PIK3CA Overgrowth Spectrum (PROS) [including - Klippel-Trenaunay syndrome and Congenital Lipomatous Overgrowth, Vascular Malformations, Epidermal Nevi, Scoliosis/Skeletal and Spinal (CLOVES) syndrome], Generalised Lymphatic Anomaly and Lymphatic and Lymphovenous malformations otherwise not classified. GLD, Generalised Lymphatic Dysplasia. Cell types: WCC, all leukocytes (white cell count); NP, neutrophils; TL, total lymphocytes; Mono, monocytes. Cell subsets were also defined by surface markers (CD2, CD3, CD4, CD8, CD16, and CD20); subsets are not mutually exclusive. CD4/8 denotes CD4 to CD8 ratio.

#### Prospective cohort

The prospective cohort of PLA patients (n=28; 16 simplex and 12 systemic; median age 37, range 15-61; 16F:12M; **Table S1**) were recruited from the Paediatric and Primary Lymphoedema Clinic at St George’s Hospital alongside an age-matched healthy control cohort (n=20; median age 51, range 23-83; 12F:8M; **Table S1**) for blood sampling to further define leukocyte subset abnormalities with additional immunophenotyping. All but 3 (n=25) had already been included in the retrospective group. All study procedures were conducted according to the principles of the Declaration of Helsinki. All participants gave written informed consent following protocols approved by the national research ethics service (NRES London 10/H0803/102 and 13/LO/1621). Not every data point for every parameter was available for every patient; numbers for each analysis are shown in the legends.

#### Clinical classification

In order to correlate laboratory findings with specific clinical entities, patients and subjects were classified according to the St George’s classification algorithm for primary lymphatic anomalies (**Figure S1**) (9). Subjects were further divided into ‘Simplex’ and ‘Systemic’ disease categories: systemic subjects were defined by a history of PLA with either current or previous episodes of pleural or pericardial effusion, ascites, intestinal lymphangiectasia or foetal hydrops, or those with a phenotype known to be associated with systemic involvement; simplex patients were those with no such internal involvement (**Tables 1**, **S1**). Data from patients with Emberger syndrome (*GATA2 deficiency*; OMIM#614038) are shown for comparison (**Table 1**) but were excluded from further analyses in this manuscript as they are known to have a primary haematopoietic disorder manifesting primarily as monocytopaenia and their unique pattern of haematological abnormalities has been described extensively elsewhere (16, 17).

Two further clinical criteria were tested for an association with lymphopaenia: the presence of genital oedema and intestinal lymphangiectasia using retrospective case-notes review data. Due to the difficulty in diagnosis and lack of consensus on diagnostic criteria for intestinal lymphangiectasia, we generated a novel predictive scoring system based on four known associated parameters: hypogammaglobulinaemia, hypoalbuminaemia, raised faecal alpha-1-antitrypsin (A1A) level, and improvement of symptoms with a medium-chain triglycerides (MCT)/low-fat diet. (Blood lymphopaenia, although a known association, was excluded being a dependent variable.) Since not all subjects had all parameters, the sum of positive variables was adjusted for incomplete data (**Table S2**) to derive ‘unlikely’, ‘possible’ and ‘probable’ descriptors. No participants had documented intestinal endoscopy or biopsy, but six subjects designated ‘probable’ on scoring had imaging results consistent with intestinal lymphangiectasia (ultrasound, CT-scan or MRI), supporting the attributed score.

### Immunophenotyping

Peripheral blood mononuclear cells (PBMCs) separated by Ficoll gradient centrifugation from prospective cohort subjects and healthy controls were analysed by multi-parameter flow cytometry (Cytoflex-S, Beckman Coulter, High Wycombe, UK) using two antibody panels: one for markers of differentiation and skin-homing, and one for proliferation and activation (**Table S3**). The gating strategy is shown in **Figure S2**. Analysis of flow cytometry plots, including t-distributed Stochastic Neighbour Embedding (t-SNE), was performed using FlowJo (BD Life Sciences. Ashland, USA).

### Statistics and data analysis

Subject group comparisons were made by ANOVA with *post-hoc* testing (Tukey) for multiple comparisons (GraphPad Prism v9, GraphPad software, San Diego, USA). Logistic regression was performed in SigmaPlot 11 (SYSTAT Software, Palo Alto, USA).

## Results

### Patients with PLA have low circulating lymphocyte counts – more so with ‘systemic’ disease

**Figure 1:**
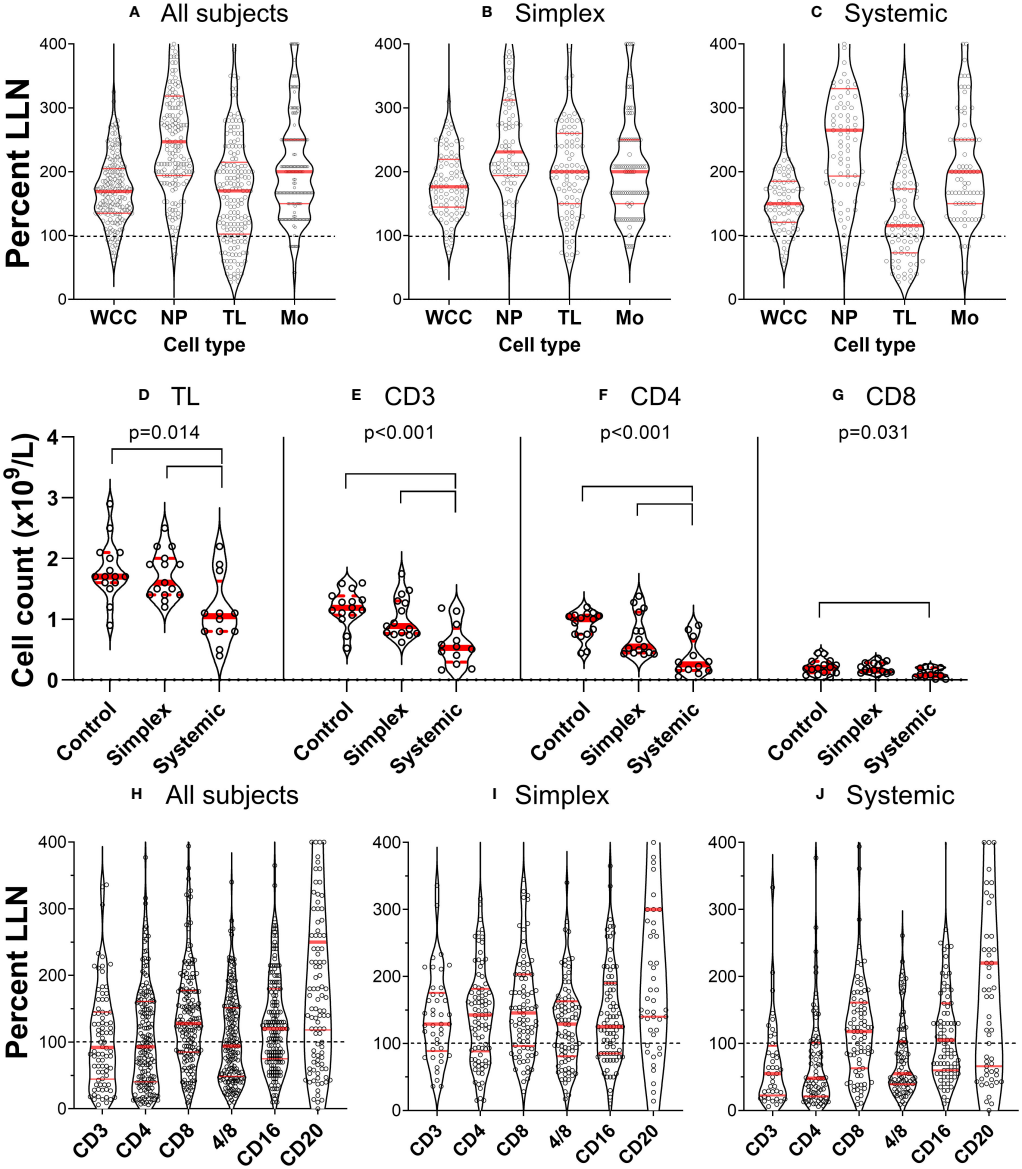
Primary lymphatic anomalies are associated with depletion of specific leukocyte subsets. **(A–C)** violin plots of cell count from individual patients in the retrospective cohort (n=177) expressed as a percentage of the lower limit of normal (LLN) for that cell type. Each point represents the result from a single participant. Red horizontal lines represent median (thick line) and 1^st^ and 3^rd^ quartiles (thin lines) for each subset; dashed line represents LLN. Data are shown for all subjects (left panel), patients with Simplex phenotypes (centre panel) and patients with Systemic phenotypes (right panel). Cell counts are: all leukocytes (white cell count, WCC); neutrophils (NP); total lymphocytes (TL); and monocytes (Mono). **(D–G)** corresponding data from the prospective cohort expressed as absolute counts based on clinical laboratory absolute lymphocyte count and subset analysis by flow cytometry (n=15 control, 15 simplex, 12 systemic – not the whole cohort as some participants did not have a quantitative cell count performed). Data are shown for **(D)** total lymphocytes (TL), **(E)** T cells (CD3^+^), and **(F)** CD4^+^ and **(G)** CD8^+^ T cell subsets. P values represent results of a one-way ANOVA between groups (control, simplex, systemic) within that cell subset, and bars show differences with multiplicity-adjusted P<0.05 by Tukey’s test. Frames **(H–J)**, plots correspond to [**(A–C)**, prospective cohort], but defined by surface marker-expression.

When we investigated white blood cell counts in patients with PLA in both retrospective and prospective groups, we found that many had low lymphocyte counts. This was not part of a generalised leukopaenia as total white blood cell counts were relatively well preserved (**Figure 1A**); different cell types were depleted differentially. To compare the impact on different cell types, data were expressed as ‘proportion of values below the lower limit of normal’ (%LLN) using the appropriate range for each cell type. By definition, about 2.5% of individuals in the general population would be expected to be in this range. This was approximately true for neutrophils and monocytes (2.9% and 5.9% respectively) but for lymphocyte counts, 22% of the whole retrospective cohort had total lymphocyte counts (TL) below the lower limit of normal (LLN) (**Figure 1A**). Subdividing patients with simplex or systemic PLA, it became clear that the major deficit was found in patients with systemic involvement (**Figures 1B, C**), where 36% of subjects had values <LLN (versus 9% in simplex subjects), a finding corroborated in the prospective cohort, where subjects with systemic disease had reduced total lymphocyte counts (**Figure 1D**, P=0.004).

### Lymphocyte depletion in PLA primarily affects CD4 cells

Further investigation of lymphocyte subsets in both retrospective and prospective groups demonstrated that CD3^+^ (T cells) were most affected, especially, but not only in those with systemic disease. Furthermore, within the CD3^+^ cell pool, CD4^+^ cell counts were most reduced, 52% of all PLA subjects in the retrospective cohort falling below the LLN; relative preservation of CD8^+^ cells resulted in reduced CD4/CD8 ratios in a substantial proportion of participants (**Figures 1H–J**) across several diagnostic categories (**Table 1**). The depletion of CD3^+^ and CD4^+^ cells was most marked in patients with systemic involvement (**Figures 1H–J**), 70% of whom had values <LLN (vs 33% of simplex subjects) in the retrospective cohort. Similarly in systemic patients in the prospective cohort, the median CD3 count was about half, and the CD4 count about a quarter of control values (**Figures 1E, F**). In contrast, CD20^+^ (B cells) and CD16^+^ (predominantly NK cells) were far less affected, being relatively preserved (**Figures 1H–J**). We also noted a relative increase in the proportion of CD4^-^CD8^-^CD3^+^ “double-negative” T cells (DN) in systemic PLA (P=0.026, *post-hoc* Tukey’s test; **Figure S3**), although this became a non-significant trend when expressed as absolute cell numbers (accounting for reduced total lymphocyte numbers).

### Patterns of CD4 lymphopaenia are disease-specific

**Figure 2:**
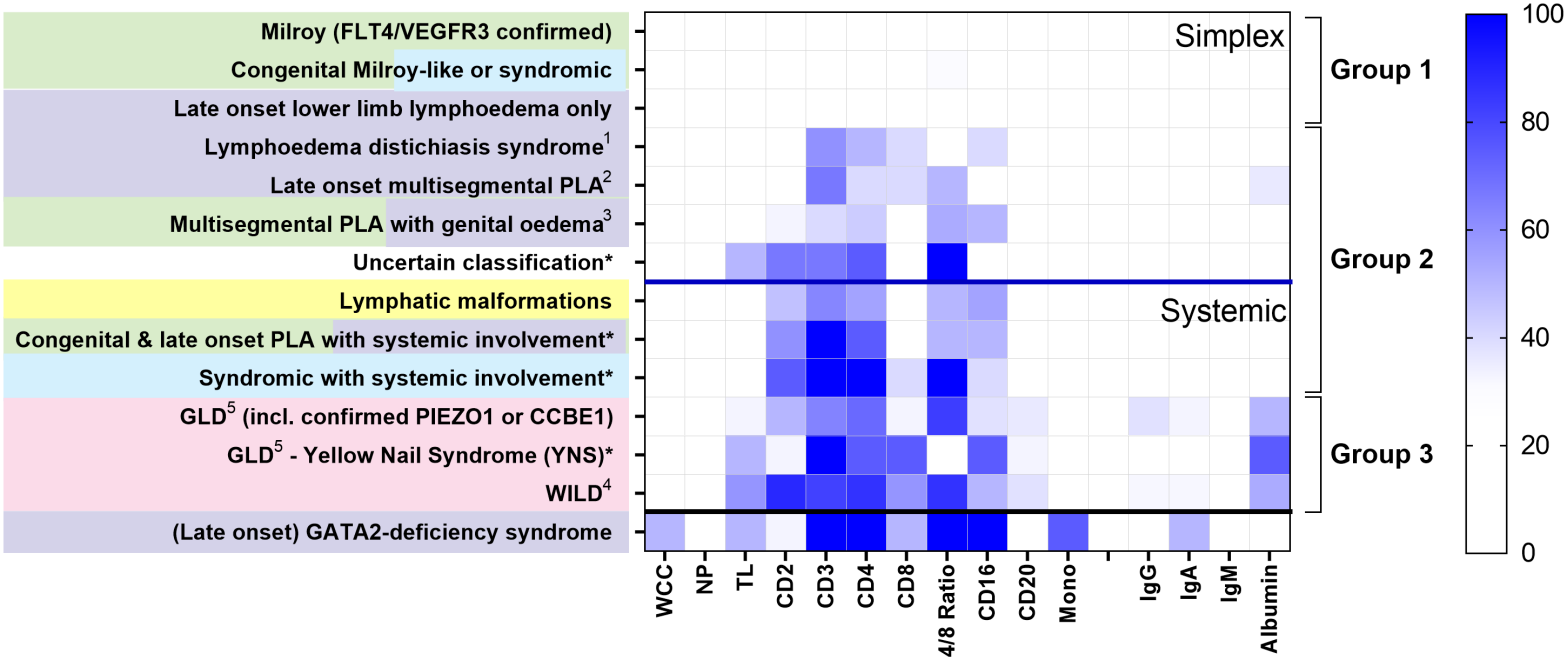
Different patterns of cytopaenia and hypoproteinaemia are associated with different clinical syndromes in patients with primary lymphatic anomalies. Subjects in the retrospective cohort were classified according to the St George’s classification algorithm for primary lymphatic anomalies (**Figure S1** - using the same colour code). The numbers in each category are shown in **Table 1**; * indicates groups with <10 participants. Annotations indicate: ^1^ *FOXC2* confirmed; ^2^ including confirmed *GJC2*; ^3^ Congenital and late onset including genital oedema - excluding *GATA2* deficiency; ^4^ Warts, Immunodeficiency, Lymphatic Dysplasia syndrome; ^5^ GLD, Generalised Lymphatic Dysplasia. For each grouping, cell and protein values numbers were expressed as the proportion of subjects with a value for that parameter less than the lower limit of normal (%<LLN), depicted as a colour scale - darker blue denoting greater percentage below LLN (see legend strip on the right). Cell types are: all leukocytes (white cell count, WCC); neutrophils (NP); total lymphocytes (TL); monocytes (Mono); alongside cells positive for specific surface markers (CD2, CD3, CD4, CD8, CD16, CD20; cell descriptors are not mutually exclusive). Proteins include: IgG, immunoglobulin G; IgA, immunoglobulin A; IgM, immunoglobulin M, and albumin. Clinical syndromes were clustered into Group 1, those without lymphopaenia or hypoalbuminaemia (>50%); Group 2, those with lymphopaenia only; Group 3, those with both lymphopaenia and hypoalbuminaemia. Categorisation into Simplex and Systemic groups is also shown as upper and middle panel; subjects with GATA2-deficiency are shown in the lowest panel for comparison.

Loss of CD4^+^ T cells is not a generalised feature of all PLA; patterns differed between clinical entities (**Figure 2**). Some had no major cellular deficits despite significant lymphoedema (Group 1, **Figure 2**). Some diagnostic groups show a pattern characterised by low CD3 and CD4 expression, with some reduction in CD2 and/or CD16, but relative preservation of CD8 and CD20, and with normal immunoglobulin and albumin levels, suggesting a common pathology (Group 2, **Figure 2**). Other diagnostic groups of PLA patients are similar to this but also present with hypoalbuminaemia (Group 3, **Figure 2**) although patients with WILD syndrome also present with more CD8 depletion and patients with Yellow Nail Syndrome (YNS), another discrete PLA entity, had a marked loss of CD8 and CD16. Patients with *GATA-2* deficiency are shown for contrast with their distinct pattern of profound monocytopaenia, as discussed elsewhere (16, 17) and are not included in subsequent analyses herein.

### The presence of systemic disease, genital oedema and intestinal lymphangiectasia independently predict CD4 lymphopaenia

**Figure 3:**
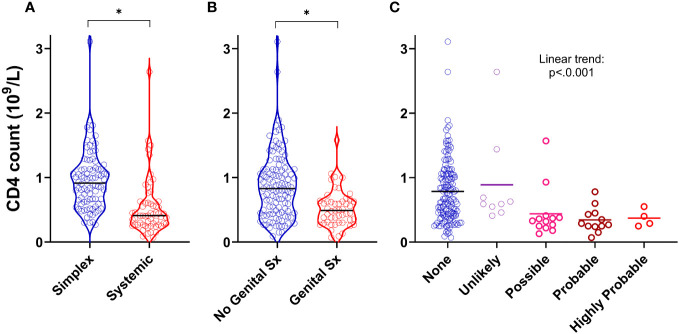
Clinical predictors of CD4 lymphopaenia in primary lymphatic anomalies. Violin plots of CD4 cell counts from retrospective cohort subjects with **(A)** systemic disease (n=59) versus simplex disease (n=86), **(B)** with genital involvement (n=49) versus no genital involvement (n=113), and **(C)** with likely intestinal lymphangiectasia scored as described in Methods (n=145, 9, 13, and 16 for None, Unlikely, Possible and Probable respectively – **Supplementary Table S2**). For **(A, B)** * P<0.0001 by Mann-Whitney test; for **(C)**, P<0.001 for linear trend by ANOVA.

We next asked whether specific clinical features (rather than diagnostic categories) were associated with CD4 lymphopaenia in PLA in both the retrospective and prospective groups. In addition to the observation that systemic disease was a strong predictor of reduced CD4 counts (**Figures 1**, **3A**), we also found that the presence of genital oedema (found in 49/162 participants – 15 missing data) was a strong predictor of CD4 lymphopaenia regardless of simplex or systemic grouping (**Figure 3B**). Furthermore, increasing likelihood of intestinal lymphangiectasia had a significant trend with reduced CD4 count (**Figure 3C**). Combining these three factors together with age and sex in a logistic regression model, all five factors (including greater age and male sex) independently increased the odds ratio of a low CD4 count (**Table S4**; n=145). Notably, having systemic or genital oedema or intestinal lymphangiectasia each increased the odds ratio more than four-fold. Chylous effusions were found in 22 patients. We considered including this in our model but noted that, of these, 21 (95%) had co-existent intestinal lymphangiectasia or genital oedema. In view of this covariance, presence of chylous effusions was not analysed separately as an independent predictor of low CD4 count.

### Naïve CD4 T cells are depleted to a greater extent than more mature differentiated cells – this skewing of the differentiation profile occurs primarily in patients with systemic disease

**Figure 4:**
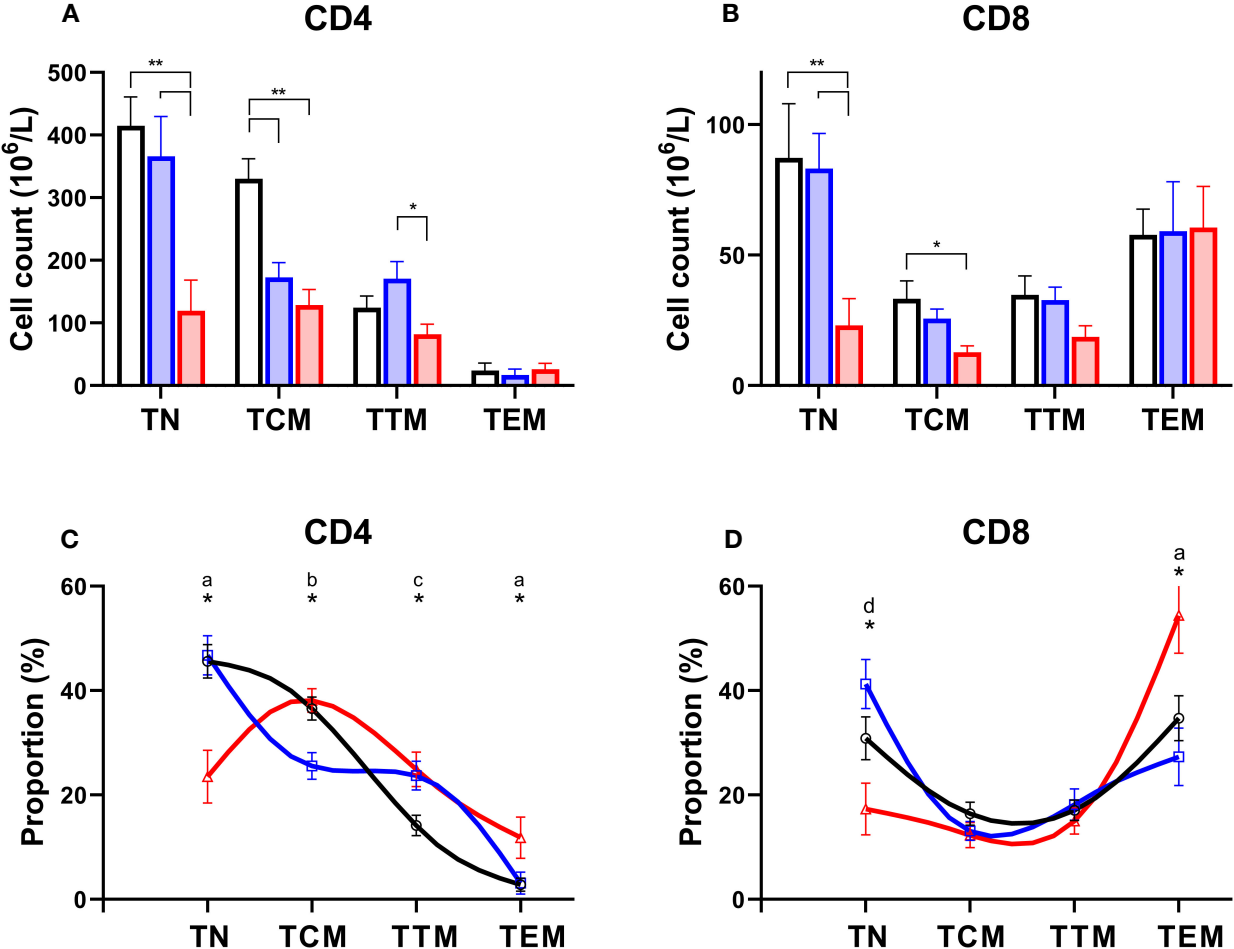
Preferential depletion of naïve T cells in primary lymphatic anomalies. Upper frames: Cell counts in absolute values (mean ± SEM, x10^6^/L) for CD4^+^ **(A)** and CD8^+^ **(B)** subsets of T cells in controls (black, n=15), patients classified with simplex disease (blue, n=15) and those with systemic disease (red, n=12). Subjects are from the prospective cohort; numbers are less than the full cohort as some subjects lacked an absolute lymphocyte count. * P<0.05; ** P<0.005 by ANOVA; bars show significant differences by Tukey’s multiple comparisons test. Lower frames: T cell subset distribution profile based on proportions of subsets as a percent of total CD4^+^ **(C)** and CD8^+^ **(D)** T lymphocytes. Subjects are from the prospective cohort and comprise controls (black line, n=20), simplex disease (blue line, n=16), and systemic (red line, n=12) categories. Values are mean ± SEM and represent relative proportions, not absolute numbers, so that within each subject group the proportion of T cell subsets sums to 100%. * indicates groups differ by ANOVA (p<0.05). Patterns of differences by *post-hoc* Tukey’s test, significant differences (P<0.05): a, C vs Sys and Sim vs Sys; b, C vs Sim and Sim vs Sys; c, C vs Sim and C vs Sys; d, Sim vs Sys only. TN, naïve cells; TCM, central memory cells; TTM, transitional memory cells; TEM, effector memory cells.

Normally T-lymphocytes follow a well-defined differentiation pathway. Naïve cells released from the thymus circulate with minimal proliferation (18), awaiting T cell receptor (TCR) engagement. Activation triggers proliferation and the generation of “memory” cells (19). Memory cells follow a sequential linear differentiation pathway through central memory cells (TCM), and transitional memory cells (TTM), to become effector memory cells (TEM); each stage is characterised by a specific phenotype. When we examined the degree to which different subpopulations were depleted in PLA subjects we found a dramatic depletion of naïve cells, and to a lesser extent TCM, in both the CD4 and CD8 compartment in patients with systemic syndromes (**Figures 4A, B**). In addition, we noted depletion of CD4^+^ TCM in simplex patients (**Figure 4A**). The consequence was a skewing of both the CD4 and the CD8 differentiation profile towards more mature differentiated cells in systemic PLA when cell counts were analysed as a proportion of the parent population (**Figures 4C, D**). In simplex PLA, although the differentiation profile was pushed to the right (more differentiated), a different pattern was seen in which CD4^+^ TCM appeared to be pushed into the TTM compartment (**Figure 4C**).

**Figure 5:**
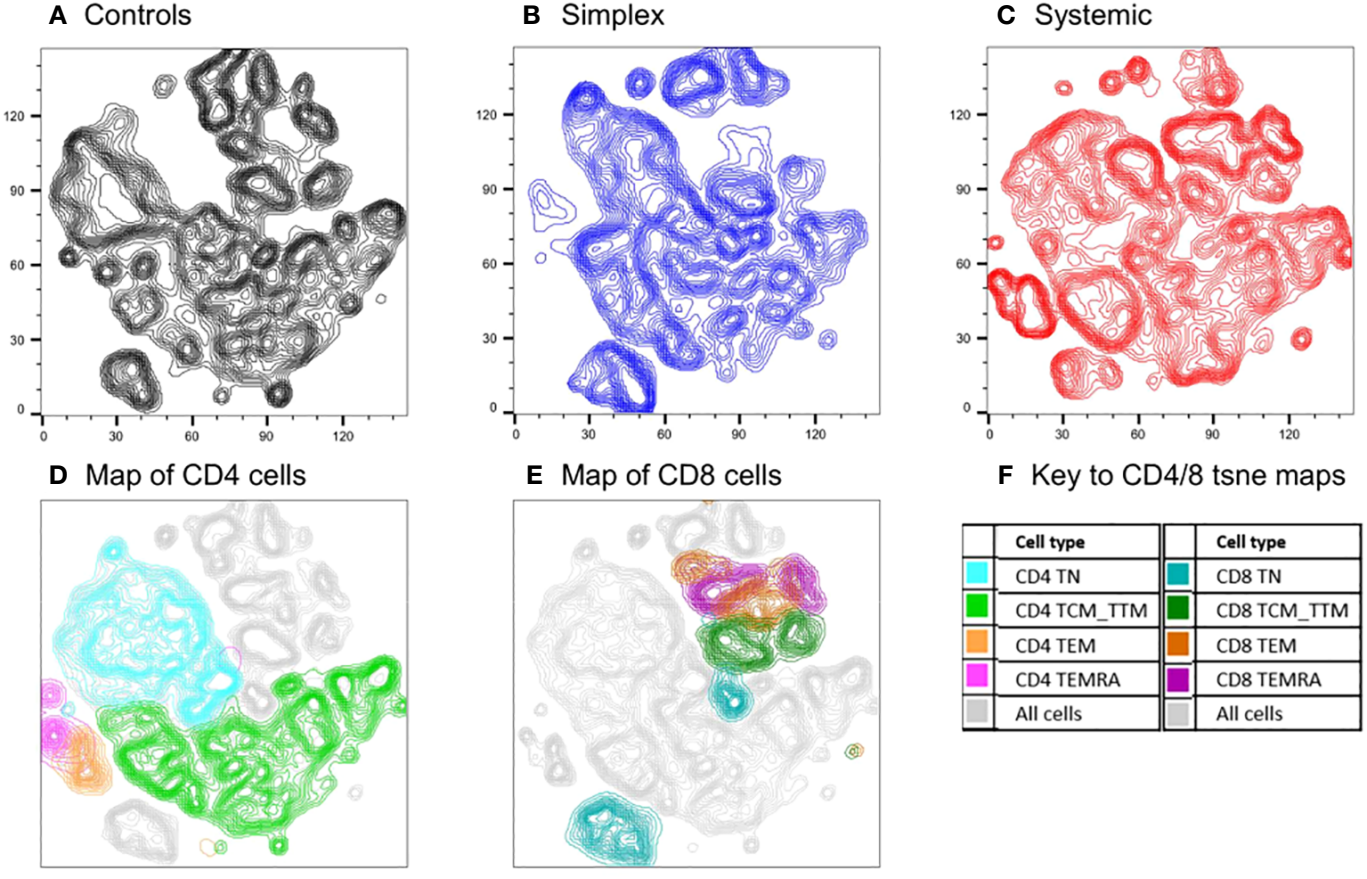
Comparison of cell populations between subjects with PLA and controls using an unbiased clustering approach (t-SNE). Map of T cell subpopulations using unbiased association mapping (t-SNE). Data were compiled by concatenating flow parameters from 20,000 cells from each of six subjects randomly selected from the simplex and systemic disease groups (n=18 in total including controls) in the prospective cohort. The map was developed using all 360,000 cells. Cell densities are shown as contour plots for control subjects **(A)**, simplex patients **(B)**, and systemic patients **(C)**; plots **(D)** and **(E)** show all cells from all subjects (grey contours) with the location of CD4 and CD8 subsets respectively highlighted by colours **(F)**.

The analysis above assumes an *a priori* hierarchical categorisation of T cell subsets. Alternative, unbiased approaches cluster cells in two-dimensions using all parameters. We used one such approach, t-SNE (t-distributed Stochastic Neighbour Embedding), to compare cell populations in PLA subjects with and without systemic disease using concatenated data from multiple subjects (n=18, including controls). The deficits in specific populations are readily apparent on these representations (**Figure 5**). Back-gating allows the characterisation of specific domains within the t-SNE plot. The loss of naïve cells, especially CD4^+^ naïve cells, in systemic PLA patients is striking with a corresponding accumulation of highly-differentiated cells not seen in controls. Interestingly, as for the differentiation profiles, changes in simplex subjects were not simply an intermediate pattern to those seen in systemic subjects – simplex subjects appeared to have their own pattern of depleted and enhanced subpopulations (**Figure 5B** versus **5A** and **5C**).

### Systemic PLA increase the proportion of naïve CD4 cells in cell-cycle but have little effect on programmed cell death

**Figure 6:**
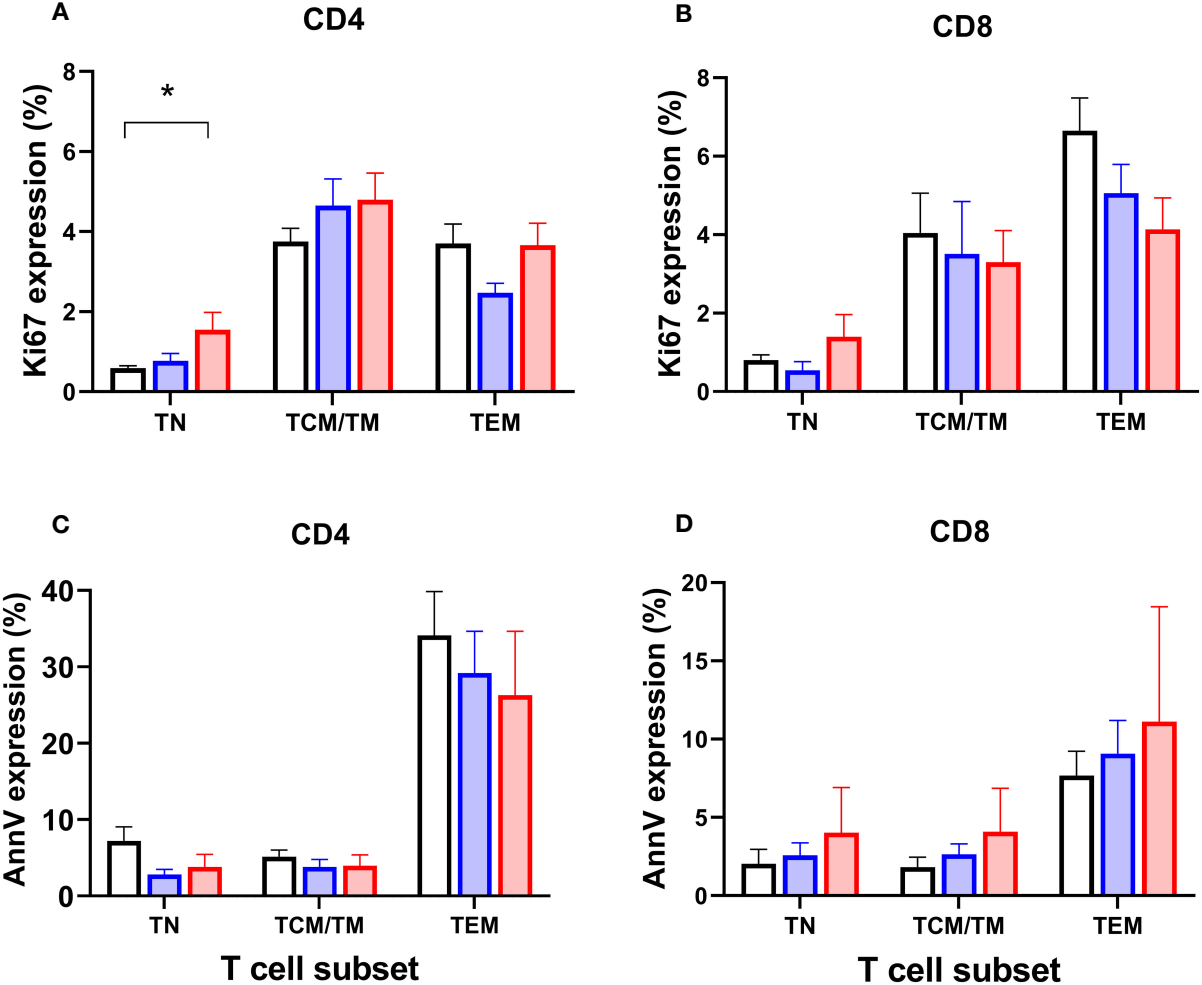
Markers of proliferation (Ki67) and apoptosis (Annexin V) in circulating T cell subsets. Proportions of CD4^+^ and CD8^+^ T lymphocytes expressing Ki67 **(A, B)** and Annexin V **(C, D)** in circulating T cell subsets in controls (black columns, n=19), patients classified with simplex disease (blue columns, n=16), and those with systemic disease (red columns, n=12) from the prospective cohort. TN, naïve cells; TCM/TM, central memory cells and transitional memory cells combined; TEM, effector memory cells. Values are mean and SEM. * P=0.015 by ANOVA; subgroup differences are significant for CD4 TN between Control vs Systemic cohorts only (*post-hoc* Tukey’s test).

To test the hypothesis that depletion of specific cell types might be driven by reduced proliferation, we analysed expression of Ki67, a marker of recent cell division (remaining positive for ~1-2 days post-mitosis) in the prospective group. As expected, Ki67 was strongly associated with differentiation stage - naïve cells have very low proliferation rates, for example (20) - so data were analysed by subset. We found a significant increase in Ki67 expression in CD4^+^ naïve cells in patients with systemic disease (P=0.015; **Figure 6A**; but not in CD8^+^ cells, **Figure 6B**), suggesting increased rates of proliferation within this usually quiescent cell subpopulation. We also investigated expression of the marker Annexin V, which identifies cells undergoing programmed cell death, but no significant differences were found between subgroups (**Figures 6C, D**).

### Systemic PLA are associated with an increased abundance of regulatory T cells and increased levels of activation in blood CD4 cells

**Figure 7:**
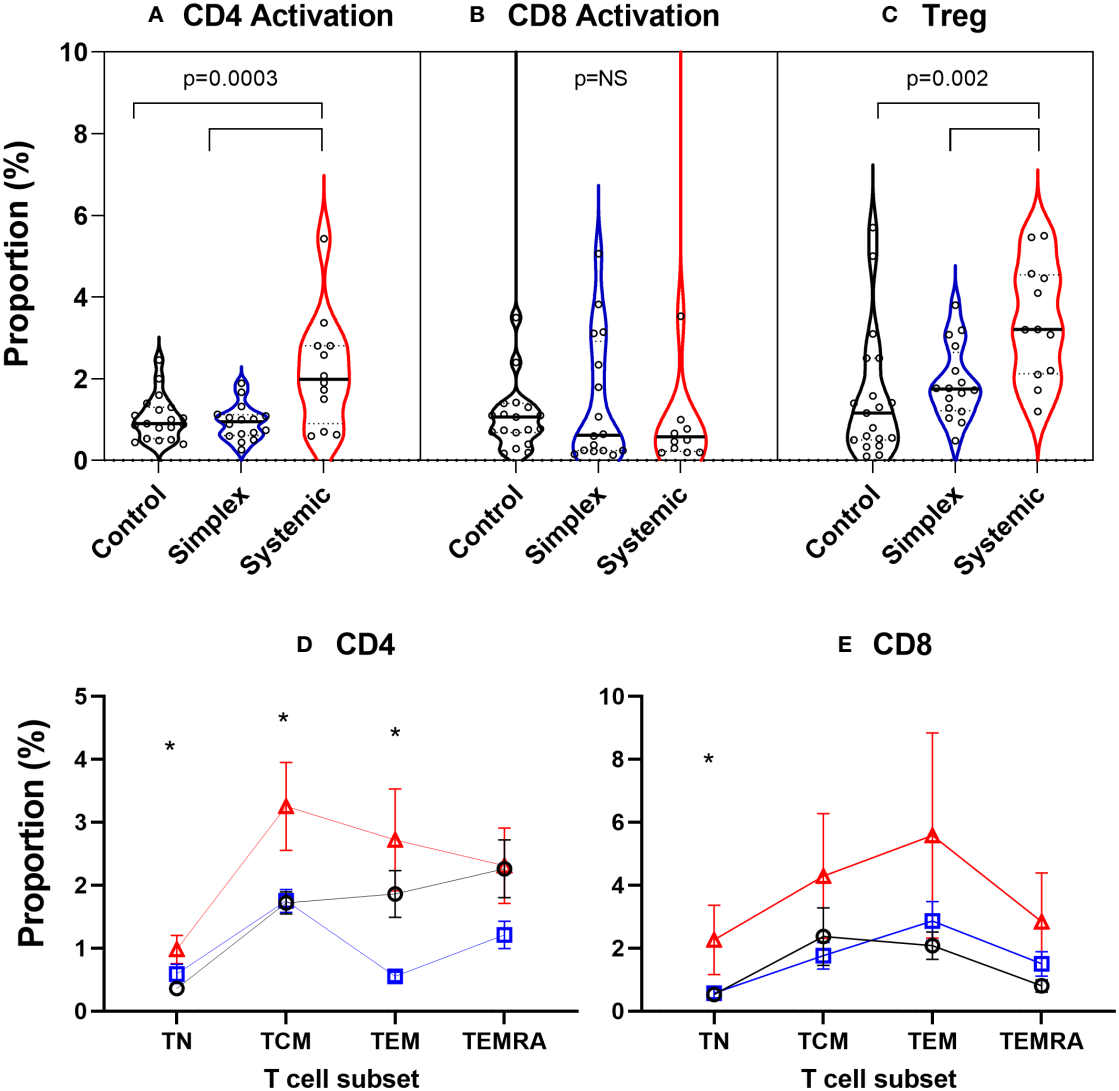
Increased proportions of activated CD4^+^ T cells and regulatory T cells in peripheral blood of subjects with systemic primary lymphatic anomalies Activated cells were defined as T cells co-expressing CD38 and HLA-DR and are shown as violin plots of proportion of CD4^+^ **(A)** and CD8^+^ T cells **(B)**. Regulatory T cells (Treg) **(C)** were defined as CD4^+^Foxp3^+^ cells. Comparisons are by ANOVA; bars show groups significantly different at P<0.05 by Tukey’s multiple comparison test. T cell subset breakdown of activation data is shown in frames **(D)** for CD4^+^ and **(E)** for CD8^+^ cells – note different scales. Subjects are from the prospective cohort and comprise controls (C, black line, n=19), patients with simplex disease (Sim, blue line, n=16) and those with systemic disease (Sys, red line, n=12). Cell subsets were defined as TN, naïve cells; TCM, central memory cells; TEM, effector memory cells; TEMRA, CD45RA expressing effector memory cells. Values are mean ± SEM. * indicates difference between groups by ANOVA (p<0.05). Subgroup differences by Tukey multiple comparison test for CD4+ are significant for TN: C vs Sys; TCM: C vs Sys, Sim vs Sys; TEM: Sim vs Sys and for CD8+ TN: p=0.042 by ANOVA but subgroup differences not significant: P=0.054 C vs Sys; P=0.073 Sim vs Sys.

In order to explore what was driving this apparent increase in proliferation in naïve CD4^+^ cells, we measured levels of activation (CD38^+^HLA-DR^+^) in the prospective group. PLA patients with systemic disease had significantly increased proportions of activated CD4^+^ cells in blood, an effect not seen in CD8^+^ T cells (**Figures 7A, B**). Since activation state is known to depend on differentiation stage, we performed subset analysis. In systemic disease patients, increased activation levels were seen not just in CD4^+^ naïve cells but also, strikingly, in central and effector memory cells (TCM & TEM; **Figure 7D**). For CD8^+^ cells, although a trend towards increased activation in the systemic disease group was observed visually, this was only significant in the naïve subset (TN; **Figure 7E**). This increased activation state was matched by an increase in the abundance of regulatory (CD4^+^Foxp3^+^) T cells (Treg) in patients with systemic disease (**Figure 7C**).

### Skin homing markers on peripheral blood T cells are unaffected by PLA

Finally, we investigated whether depletion of lymphocytes from blood corresponded with changes in expression of the skin-homing markers CCR10, CLA and CCR4 in the prospective group. As expression of these receptors is highly dependent upon differentiation state, we again performed subset analysis. We found that for both CD4^+^ and CD8^+^ cells, skin-homing markers were most often expressed in intermediate-differentiated cells (TCM and TTM) but expression levels in control subjects, and simplex and systemic PLA patients showed remarkable concordance across all three markers in all subsets (**Figure S4**). No differences between patient groups were found except that CD4-CCR10, CD8-CCR10 and CD8-CCR4 expression were suppressed in simplex patients (P=0.02, 0.04 and P<0.001 respectively by two-way ANOVA), but the biological magnitude of this effect was small.

### Hypoalbuminaemia and hypoglobulinaemia are less common than lymphopaenia and restricted to specific disease groups

In order to interpret the occurrence of cytopaenias, we also investigated the frequency of hypoalbuminaemia and hypoglobulinaemia in the retrospective cohort. For simplex patients, albumin levels <LLN were only really apparent in simplex subjects in the late onset multisegmental PLA group (**Figure 2**), but were observed in many patients with systemic involvement (Group 3, **Figure 2**). Overall, hypoalbuminaemia affected substantially fewer subjects than CD4 depletion - 29% versus 52% of all PLA subjects and 40% versus 70% of those with systemic disease, suggesting that the pathological mechanism driving CD4 lymphopaenia was more pervasive or more extensive than the mechanism driving albumin depletion. Immunoglobulins were less affected (**Figures 2**, **S4**; **Table S5**) with no apparent difference between the three classes studied (**Figure S5**).

## Discussion

This study has demonstrated that PLA patients with dysfunctional lymphatics have a selective reduction in circulating lymphocytes which preferentially affects naïve CD4^+^ T cells. The observed pattern of lymphopaenia did not appear to be clinical or genotype-specific but was shared across several primary lymphatic anomalies suggesting a common mechanism. Findings were consistently either restricted to, or more marked in, patients with systemic disease (including intestinal lymphangiectasia), or to those with evidence of genital oedema. Genital oedema was strongly associated with CD4 lymphopaenia, probably reflecting extensive disturbed lymph drainage in affected patients. Exceptions to this general pattern of cytopaenia included YNS, WILD and generalised lymphatic dysplasia (GLD), where patterns more suggestive of generalised cell loss with hypoalbuminaemia were observed (Group 3 in **Figure 2**). As expected, a unique signature was seen in *GATA2*-deficiency (Emberger syndrome) where monocytopaenia results from a distinct haematological syndrome (21); this pattern was not further investigated in this study.

In terms of the likely mechanism of lymphopaenia in PLA, broadly speaking, depletion of a cell subset, within an anatomical compartment such as the blood, results either from reduced production or increased loss. In terms of production, our Ki67 data (**Figure 6**) do not support the hypothesis that depletion results from reduced proliferation. Indeed, the opposite appeared to be the case – the most depleted cells (naïve CD4^+^ T cells), had higher, not lower, rates of proliferation. Further studies measuring proliferation directly by *in vivo* labelling would clarify the true extent of this response.

Loss of cells from blood is thus the most likely mechanism. ‘Loss’ might consist of accelerated cell death, redistribution out of the body altogether, migration to another anatomical compartment, or phenotype change (so the cell is counted as a different type of cell). Our Annexin V data suggests that lymphocyte depletion does not result from accelerated cell death, although the power of the Annexin data to detect small changes in death rates may be limited. Total loss from the body would occur primarily through the intestine. The resemblance of our cellular phenotypic findings to those in primary and secondary intestinal lymphangiectasia is striking (11–14). One interpretation of this similarity is that subclinical intestinal lymphangiectasia is far more prevalent in PLA than currently appreciated. Evidence against this interpretation includes the observations: (i) that most of our participants with CD4 lymphopaenia had no other features to suggest intestinal lymphangiectasia and, (ii) that lymphopaenia was considerably more prevalent in PLA patients than hypoalbuminaemia or hypoglobulinaemia - generally considered hallmarks of intestinal lymphangiectasia, (**Figures 2**, **S5**; **Table S5**). An alternative interpretation, which we consider more evidence-based, is that intestinal lymphangiectasia, along with genital oedema (**Figure 3**) and the presence of chylous effusions, which are often found in the same participants, all represent surrogate clinical markers for severe generalised lymphatic dysfunction, rather than representing the direct pathological mechanism for CD4 depletion. Of course, the two are not mutually exclusive.

Loss from the circulation by sequestration in tissues is likely to be important in PLA. Given that at any one time only ~0.5% of lymphocytes are circulating in blood (22), small variations in the 200:1 ratio between the extra-vascular and intra-vascular compartments will disproportionately affect circulating cell numbers. Increased tissue residence due to lymphatic dysfunction is likely to preferentially deplete blood naïve cells which recirculate through lymphatics more than memory cells (11, 23). Increased CD4/CD8 ratios in lymphatic fluid compared with blood suggest selective extra-vascular sequestration of CD4 lymphocytes in tissue lymphatic fluid (24, 25). CD4 lymphocyte numbers are increased in human lymphoedema biopsy samples and quantitatively correlate with disease severity (26). Impaired trafficking does not just entrap lymphocytes in tissues; neutrophils, macrophages, and dendritic cells (DC) also accumulate (27), creating a mixed inflammatory infiltrate. Local accumulation of CD4 lymphocytes further promotes lymphatic dysfunction through pro-fibrotic, peri-lymphatic inflammation and inhibited collateral vessel formation (28). Indeed CD4^+^ T cells have been shown to have a key role in driving and regulating local lymphangiogenesis and lymphatic function (8).

The correlate of increased entrapment in tissues for lymphocytes is reduced access to and reduced relative dwell times in lymph nodes. Lymph nodes have been shown to play a key role in T cell homeostasis, providing an environment within which T cells receive survival signals. Studies in mice demonstrate that blocking access to lymph nodes reduces peripheral CD4^+^ and CD8^+^ naïve T cell survival, likely through reduced access to IL-7 produced by T zone fibroblastic reticular cells (29). In patients with PLA, a similar process is likely to operate; reduced access to and residence within lymph nodes may deprive recirculating T cells of survival signals by restricting access to local environments high in IL-7; this may partly explain the relative excess depletion of naïve cells observed in our study. Furthermore, impaired homing of DC may exacerbate the situation by reducing DC-induced CCL21 production by fibroblastic reticular cells (30), further reducing lymph node dwell times.

Using multicolour flow cytometry to phenotype immune cell populations in circulating blood, we noted increased rates of activation in circulating cells in patients with systemic PLA (**Figure 7**). We consider that this likely mirrors the increased levels of T cell activation, especially for CD4^+^ cells in lymphoedematous tissues (2, 31), although increased T cell activation is also seen in other human lymphopaenic conditions, where it may reflect systemic compensatory homeostasis (32). In mice models, activation of T cells is pivotal to the development of lymphoedema (8, 26–28, 33, 34). Activated Th1 and Th17 T cells interact with macrophages to promote lymphangiogenesis (33), whilst lymph stasis due to impaired lymphatic drainage results in a CD4^+^ T cell inflammation and T-helper 2 (Th2) differentiation which in turn promotes fibrosis and lymphatic dysfunction (28). Increased T cell activation may in turn lead to greater rates of cell death (activation-induced cell death, AICD) and exhaustion, as seen in filarial lymphoedema (35, 36).

In concert with increased activation, we found increased numbers of regulatory T-cells (Treg) in blood (**Figure 7**). A parallel increase in Treg numbers in tissue has been observed in both human breast cancer-related lymphoedema and mouse experimental lymphoedema (8, 26). In lymphoedematous mice, isolation and reinfusion of Tregs, i.e. adoptive Treg transfer, reversed the lymphoedema and promoted lymphatic drainage (37). Hence the presence of Treg represents a double-edged sword – they limit the extent of lymphoedema, but also contribute to local and possibly systemic immunosuppression (26).

Although a clear limitation of our study is that we only analysed cells in blood, it is striking how the changes we have observed in blood mirror those seen in tissue in other human and animal studies. Further studies analysing lymphocyte subsets within lymphoedema tissue samples will enhance our understanding of the role of lymphatics in local immune homeostasis. Further immunophenotyping and more focused analysis of distinct PLA may also contribute to our understanding as our *a priori* categorisation into two broad categories, simplex and systemic, may have conflated quite disparate immune states into one as suggested by the observations of quite specific profiles for the discrete diagnostic groups YNS and WILD. Despite careful clinical phenotyping, our study was limited by difficulty grading the severity of lymphatic dysfunction. Existing grading methods (38) are prone to inter- and intra-observer variability and further studies optimising grading methodology and addressing potential associations with immunological dysfunction are needed. Any study of this nature of a complex and unusual set of diseases is likely to be limited by numbers, especially when subgroup analysis is performed, hence caution needs to be exercised in the interpretation of the results. However, this is the only large cohort study of this nature that we are aware of and the results were characterised by highly-significant P values. Clearly further studies to confirm our findings would increase confidence in our conclusions. Some of the changes we have observed in PLA, such as depletion of naïve cell numbers and increased terminally-differentiated cell numbers in blood, do mirror those seen in ageing, which occur despite relatively well-preserved homeostasis (39, 40). However the effects of PLA seen here are far more dramatic than could be attributed to ageing, are still observed when ageing is controlled for by logistic regression, and are still seen even in age-matched cohorts (**Table S1**).

The clinical significance of these observations is non-trivial. Localised immune incompetence has been assumed within areas affected by lymphoedema, but our observation of a more generalised, often profound, immune deficit has previously remained largely unrecognised. The levels of CD4 lymphopaenia seen in PLA patients resemble those seen in AIDS where CD4 counts <0.2x10^9^/L are associated with opportunistic infections. Our clinical unpublished informal observations that such infections are not typically seen in PLA patients may reflect relative preservation of tissue-resident T cells (TRM) in PLA and the presence of a stable highly proliferative naïve T cell pool. However, this combination of local and systemic immunosuppression may contribute to the risk of malignancies in PLA patients such as the aggressive angiosarcoma of Stewart-Treves syndrome (41), and other immune-related dermatological manifestations including neutrophilic dermatoses (1). The infection burden in this cohort deserves further study as it is not well documented, but clinicians working with PLA patients recognise considerable morbidity due to recurrent and recalcitrant viral warts and episodes of cellulitis (42). Our own clinical observations strengthen the case for prompt and active treatment of such infections. Furthermore, our findings may have implications for other, more common settings where lymphatic dysfunction may occur such as malignancy, obesity (34, 43) and ageing (44) where cytopaenias may be under-recognised.

## Conclusion

Our findings suggest that patients with PLA have a more generalised immune deficit than previously recognised. The presence of systemic disease, genital oedema and intestinal lymphangiectasia independently predict severity. The abnormal patterns of immune cell phenotype described are instructive for understanding local immune homeostasis, are clinically relevant, emphasising the need for prompt intensive treatment of infections, and may have implications for other conditions where more subtle lymphatic dysfunction may disturb immune homeostasis.

## Data availability statement

The raw data supporting the conclusions of this article will be made available by the authors, without undue reservation.

## Ethics statement

The studies involving humans were approved by National Research Ethics Service - London (refs 10/H0803/102 and 13/LO/1621). The studies were conducted in accordance with the local legislation and institutional requirements. Written informed consent for participation in this study was provided by the participants, or their legal guardians/next of kin.

## Author contributions

JP: Conceptualization, Data curation, Formal Analysis, Methodology, Validation, Writing – original draft, Writing – review and editing, Investigation. LH: Data curation, Formal Analysis, Investigation, Validation, Writing – original draft, Writing – review and editing, Project administration. SM: Data curation, Project administration, Validation, Writing – review and editing, Conceptualization, Funding acquisition, Resources. MZ: Project administration, Resources, Writing – review and editing, Investigation. SJ: Resources, Writing – review and editing, Data curation, Conceptualization. KG: Data curation, Resources, Writing – review and editing, Validation, Funding acquisition. PO: Data curation, Resources, Validation, Writing – review and editing, Conceptualization, Funding acquisition, Supervision. PM: Conceptualization, Funding acquisition, Resources, Validation, Writing – review and editing, Supervision. DM: Conceptualization, Data curation, Methodology, Validation, Writing – review and editing, Formal Analysis, Funding acquisition, Supervision, Writing – original draft, Investigation.
