# Supplementary material for: Profound and selective lymphopaenia in primary lymphatic anomaly patients demonstrates the significance of lymphatic-lymphocyte interactions
Source: Front Immunol. 2023 Nov 3;14:1279077. doi: 10.3389/fimmu.2023.1279077 (PMC10656747; doi:10.3389/fimmu.2023.1279077)
Supplement: Supplementary file 1 [file DataSheet_1.pdf]

Supplementary Material

Profound and selective lymphopaenia in Primary Lymphatic Anomaly patients demonstrates the significance of lymphatic-lymphocyte interactions

Julian Pearce, Linda Hadcocks, Sahar Mansour, Malou van Zanten, Steve Jeffery, Kristiana Gordon, Pia Ostergaard, Peter Mortimer, Derek C. Macallan *

* **Correspondence:** Derek C. Macallan: macallan @sgul.ac.uk

Contents

Table S1. Demographics and clinical classification of prospective cohort

Table S2. Scoring method for likelihood of intestinal lymphangiectasia

Table S3. Flow cytometry panels

Table S4. Multiple logistic regression for predictors of CD4 cytopaenia

Table S5. Protein levels in retrospective cohort by diagnostic category

Figure S1. Clinical Classification algorithm

Figure S2. Gating Strategy

Figure S3. Increased proportion of double-negative CD3^{+} T cells in PLA patients

Figure S4. Skin-homing receptor expression on T cell subsets in controls and PLA subjects

Figure S5. Albumin and immunoglobulin levels in PLA subjects

frontiers

Table S1. Demographics and clinical classification of the Prospective Cohort

	Ref ID	Cohort	Sex	Age
Control	ILO55	control	F	23
	ILO86	control	F	25
	ILO58	control	F	33
	DW20	control	F	41
	ILO50	control	F	48
	ILO54	control	F	53
	IA192	control	F	53
	DW25	control	F	56
	ILO57	control	F	57
	IA188	control	F	57
	IA116	control	F	68
	IA115	control	F	76
	ILO56	control	M	25
	IA189	control	M	26
	ILO53	control	M	32
	IA190	control	M	34
	ILO62	control	M	46
	IA191	control	M	61
	ILO61	control	M	67
	IA121	control	M	83
Simplex	ILO87	Milroy (*FLT4/VEGFR3* confirmed)	F	22
	ILO80	Milroy (*FLT4/VEGFR3* confirmed)	F	32
	ILO89	Milroy (*FLT4/VEGFR3* confirmed)	F	33
	ILO78	Milroy (*FLT4/VEGFR3* confirmed)	F	41
	ILO88	Milroy (*FLT4/VEGFR3* confirmed)	F	57
	ILO68	Milroy (*FLT4/VEGFR3* confirmed)	M	35
	ILO84	Lymphoedema Distichiasis Syndrome (*FOXC2* confirmed)	F	17
	ILO69	Lymphoedema Distichiasis Syndrome (*FOXC2* confirmed)	F	29
	ILO79	Lymphoedema Distichiasis Syndrome (*FOXC2* confirmed)	F	49
	ILO63	Lymphoedema Distichiasis Syndrome (*FOXC2* confirmed)	M	47
	ILO77	Lymphoedema Distichiasis Syndrome (*FOXC2* confirmed)	M	48
	ILO85	Lymphoedema Distichiasis Syndrome (*FOXC2* confirmed)	M	58
	ILO81	Late onset multisegmental PLA (*GJC2* confirmed)	F	24
	ILO82	Late onset multisegmental PLA (*GJC2* confirmed)	M	53
	ILO73	PROS (*PIK3CA* mosaicism)	F	22
	ILO83	Unknown	F	15
Systemic	ILO74	Syndromic with systemic involvement (*RIT1* confirmed)	M	26
	ILO47	GLD (incl. confirmed *PIEZO1* or *CCBE1*)	F	23
	ILO67	GLD (incl. confirmed *PIEZO1* or *CCBE1*)	F	39
	ILO49	GLD (incl. confirmed *PIEZO1* or *CCBE1*)	F	39
	ILO48	GLD (incl. confirmed *PIEZO1* or *CCBE1*)	M	29
	ILO66	GLD (incl. confirmed *PIEZO1* or *CCBE1*)	M	45
	ILO46	GLD (incl. confirmed *PIEZO1* or *CCBE1*)	M	45
	ILO75	GLD – Yellow Nail Syndrome (YNS)	F	51
	ILO76	GLD – Yellow Nail Syndrome (YNS)	M	61
	ILO65	WILD	F	24
	ILO64	WILD	M	33
	ILO70	WILD	M	37

Table S1. Subjects were sorted by diagnostic category, sex, then age. Categories follow the diagnostic algorithm in Figure S1 and correspond to those shown in Table 1. Age distributions were not significantly different between the three cohorts (P>0.05 by ANOVA).

Table S2. Scoring method for likelihood of intestinal lymphangiectasia

		Number of positive variables				
		0	1	2	3	4
Number of variables available	1	Unlikely	Possible			
	2	Unlikely	Possible	Probable		
	3	Unlikely	Possible	Probable	Probable	
	4	Unlikely	Unlikely	Possible	Probable	Probable

Table S3. Flow cytometry panels

Differentiation and homing panel

Marker	Colour/Format	Clone	Company
CD3	BV510	UCHT1	BioLegend
CD4	PerCP-Cy5.5	OKT4	BioLegend
CD8	FITC	RPA-T8	BioLegend
CD45RA	PE-Cy7	HI100	BioLegend
CD28	BV650	CD28.2	BioLegend
CCR4 (CD194)	PE Dazzle594	L291H4	BioLegend
CCR7	BV421	GO43H7	BioLegend
CLA	APC	REA1101	Miltenyi
CCR10	PE	1B5	BD
Live/Dead	APC-Cy7	FVD780	ebioscience

Proliferation and activation panel

Marker	Colour/Format	Clone	Company
CD3	BV510	UCHT1	BioLegend
CD4	PerCP-Cy5.5	OKT4	BioLegend
CD8	FITC	RPA-T8	BioLegend
CD45RA	PE-Cy7	HI100	BioLegend
CD28	BV650	CD28.2	BioLegend
CD38	PE Dazzle594	HB-7	BioLegend
HLA-DR	BV605	L243	BioLegend
FoxP3	BV421	206D	BioLegend
Ki-67	APC	Ki-67	BioLegend
Annexin V Apoptosis	PE		ebioscience
Live/Dead	APC-Cy7	FVD780	ebioscience

Table S4. Multiple logistic regression for predictors of CD4 cytopaenia

Logit P = -2.410 + (0.0275 * Age) + (1.120 * F=0;M=1) + (1.491 * Systemic) + (1.526 * Genital) + (1.560 * IL[§]=1)
Low CD4 count defined as <0.7 x 10^9/L.
[§] IL = presence of Intestinal Lymphangiectasia

N = 145 (retrospective cohort, n=177; missing data in 32 subjects)

Estimation Criterion: Maximum likelihood
Dependent Variable: CD4<LLN
Positive response (1): 1
Reference response (0): 0
Number of unique independent variable combinations: 125

Pearson Chi-square Statistic: 137.7 (P = 0.490)
Likelihood Ratio Test Statistic: 63.6 (P = <0.001)
-2*Log(Likelihood) = 137.2
Hosmer-Lemeshow Statistic: 8.66 (P = 0.372)

Threshold probability for positive classification: 0.500
Classification Table:

	**Predicted Reference**	**Predicted Positive**	**Totals**
Actual Reference Responses	55	15	70
Actual Positive Responses	15	60	75
Totals	70	75	145

Details of the Logistic Regression Equation

**Ind. Variable**	**Coefficient**	**Standard Error**	**Wald Statistic**	**P value**	**VIF**
Constant	-2.410	0.540	19.885	<0.001	
Age (per year)	0.0275	0.0130	4.504	0.034	1.005
Male sex	1.120	0.435	6.617	0.010	1.135
Systemic	1.491	0.520	8.234	0.004	1.659
Genital	1.526	0.494	9.545	0.002	1.124
IL	1.560	0.782	3.978	0.046	1.578

**Ind. Variable**	**Odds Ratio**	**5% Conf. Lower**	**95% Conf. Upper**
Constant	0.0899	0.0312	0.259
Age (per year)	1.028	1.002	1.054
Male gender	3.065	1.306	7.196
Systemic	4.443	1.604	12.304
Genital	4.599	1.747	12.106
IL	4.758	1.027	22.036

Table S5. Protein levels in retrospective cohort by diagnostic category

Group	n	Plasma Protein (%LLN)			
		IgG	IgA	IgM	Albumin
**Simplex**					
Milroy (*FLT4/VEGFR3* confirmed)	10	0	14	0	0
Congenital Milroy-like or syndromic	16	0	0	8	0
Late onset lower limb lymphoedema only	14	0	8	0	0
Lymphoedema distichiasis syndrome (*FOXC2* confirmed)	13	0	25	0	0
Late onset multisegmental PLA (incl. confirmed *GJC2*)	15	0	0	0	36
Congenital and late-onset multisegmental PLA including genital oedema (excluding *GATA2*)	20	0	11	11	27
Uncertain classification	4	0	0	0	0
**Systemic**					
Lymphatic malformations	20	0	6	11	16
Congenital and late onset peripheral lymphoedema with systemic involvement	5	0	20	20	25
Syndromic with systemic involvement	5	20	0	20	20
GLD (including confirmed *PIEZO1* or *CCBE1*)	24	38	33	17	50
GLD – Yellow Nail Syndrome (YNS)	4	0	0	0	75
Warts, Immunodeficiency, Lymphatic Dysplasia (WILD)	22	32	32	14	53
(Late onset) *GATA2*-deficiency syndrome	5	25	50	0	0

Data from the retrospective cohort of 177 subjects with primary lymphatic anomalies (PLA) from the National Primary Lymphatic Anomaly Register. Table shows subjects by diagnostic category grouped according to the Gordon et al classification (Figure S1); colours refer to the colour code in that classification (Figure S1); genotypes are given where known. GLD, Generalised Lymphatic Dysplasia. Subjects with *GATA2*-deficiency syndrome are shown for comparison. Data show number of subjects in each diagnostic category (n) and the percentage of those subjects with a protein concentration below the lower limit of normal (%LLN) for each protein, including Immunoglobulin G (IgG); Immunoglobulin A (IgA); Immunoglobulin M (IgM); and albumin.

Figure S1. Clinical Classification algorithm

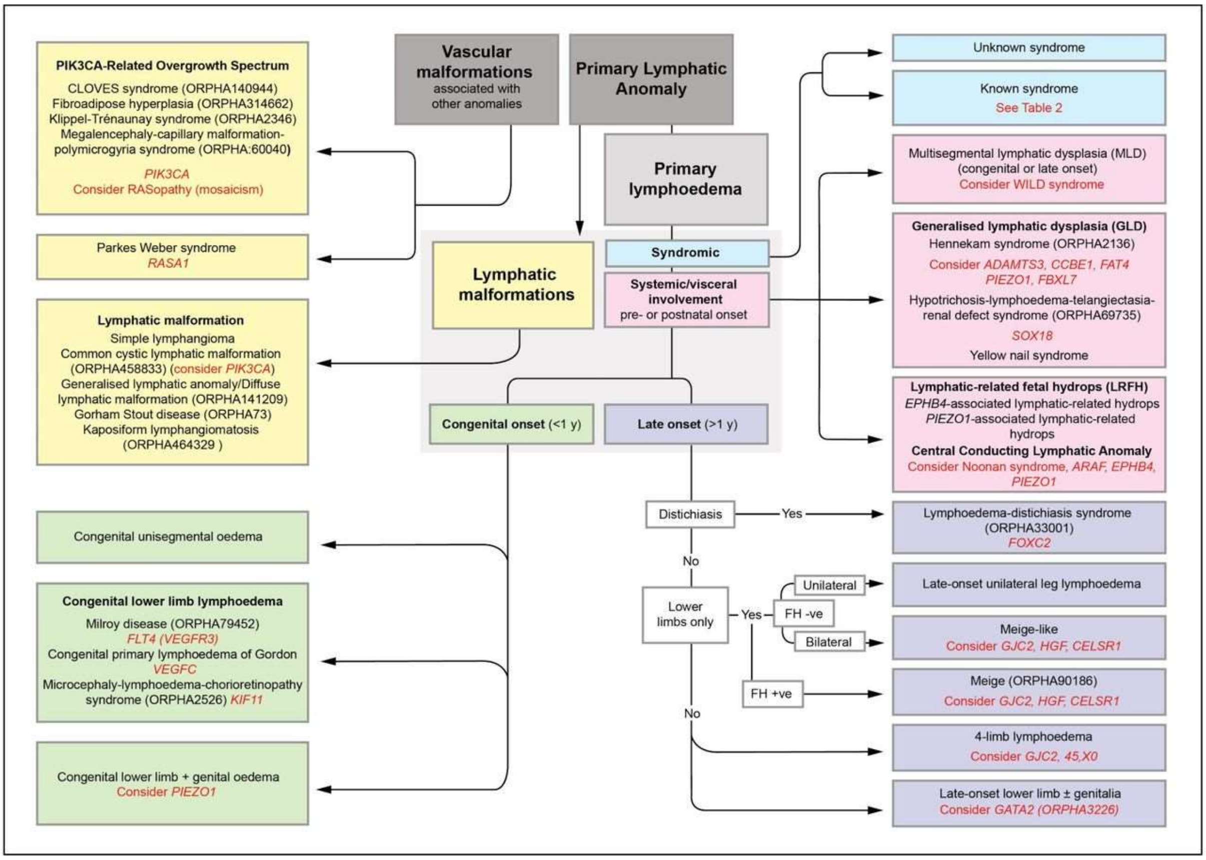

The classification algorithm for primary lymphatic anomalies (PLA) used at St George's Hospital. Patients and subjects included in the study were classified using the five main groupings which are colour coded with their various clinical subtypes of disease. Many of the individuals further classified with 'systemic' disease would fall within the pink section of the algorithm. Text in red indicates the suggested genetic test or differential diagnosis for the subgroup. FH, family history; +ve, positive; −ve, negative. (Image shared by St George's Lymphovascular Research Group under the CC BY-SA 4.0 International licence on Wikimedia Commons).

Figure S2. Gating Strategy

Illustration of gating strategy taking CD4⁺ T cells as an example. Similar analyses were performed for CD8⁺ T cell subsets.

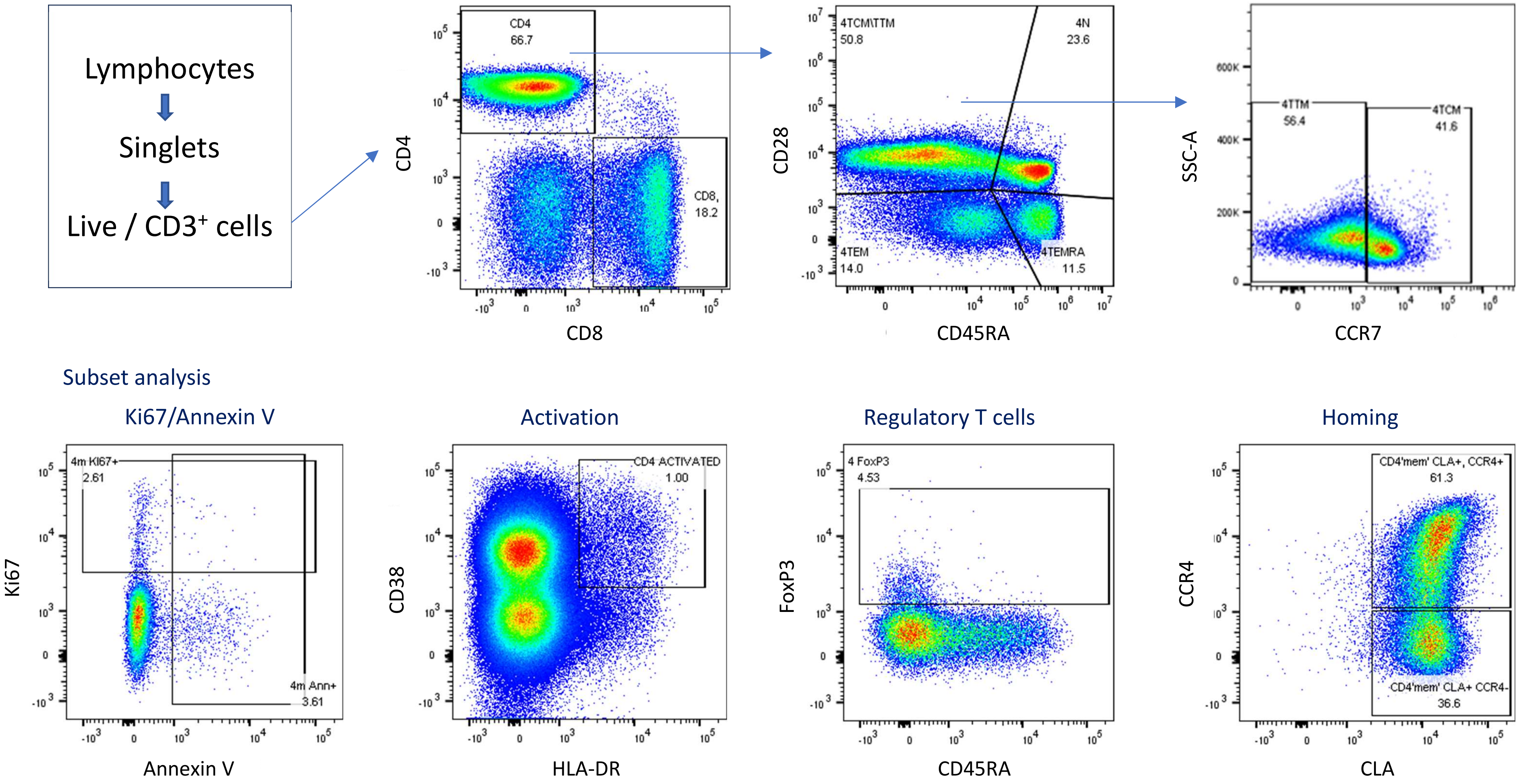

Figure S3. Increased proportion of double-negative CD3⁺ T cells in PLA patients

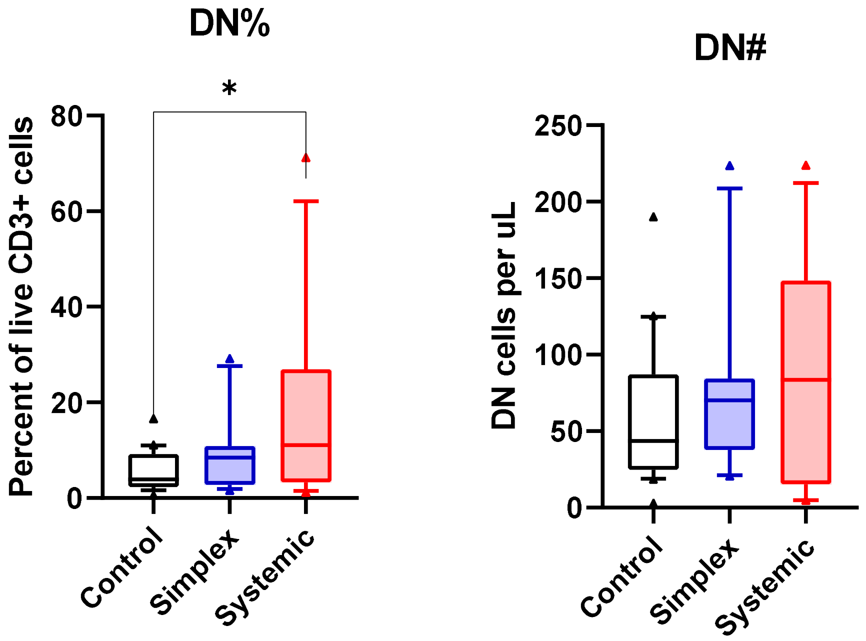

Data are shown for the proportion of circulating live $CD3^{+}CD4^{-}CD8^{-}$ 'double-negative' cells in subjects in the prospective cohort, shown either as proportion of all $CD3^{+}$ cells (a); or as absolute numbers (b). Box and whiskers are means with 10-90% plots for whiskers; triangles are outlier values. * P=0.026 by ANOVA with Tukey's post-hoc test (P=0.020).

Figure S4. Skin-homing receptor expression on T cell subsets in controls and PLA subjects

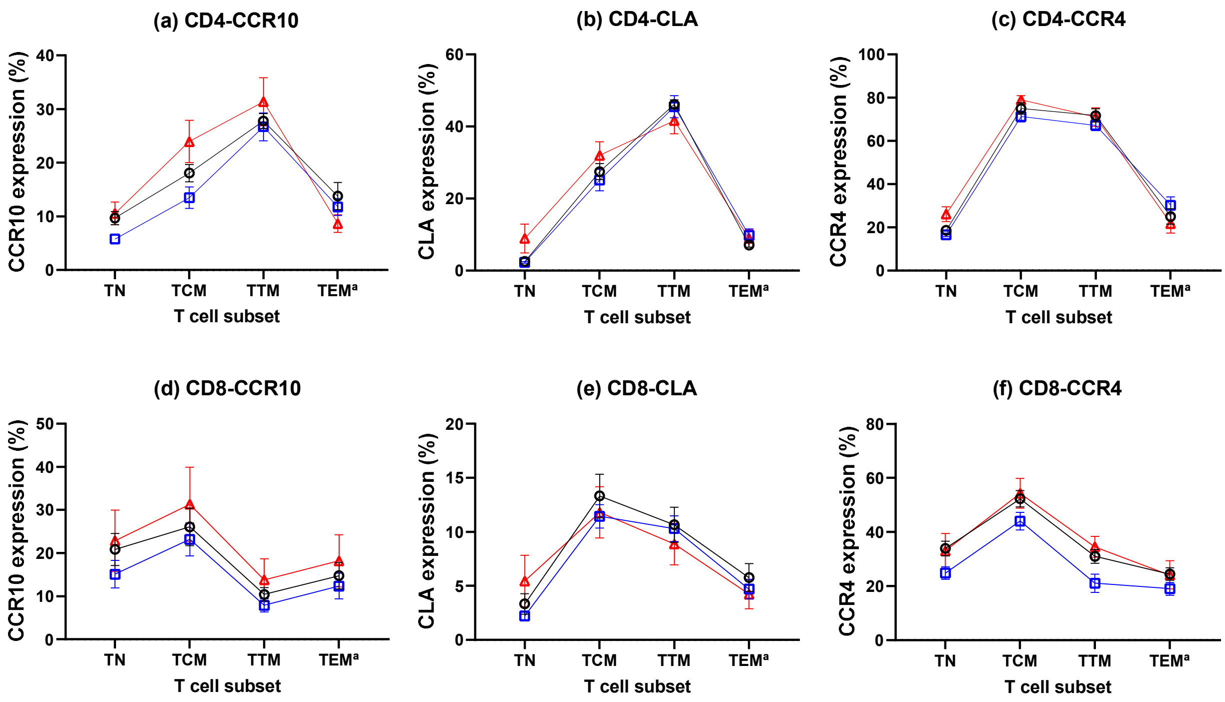

Values are mean and SD of expression rates of CCR10, left panels; CLA, middle panels; and CCR4, right panels, by prospective patient subgroup with $CD4^+$ in top panels and $CD8^+$ in lower panels. TN, naïve cells; TCM, central memory cells; TTM, transitional memory cells; TEM^a is effector memory cells (TEM) and re-expressing effector memory cells (TEMRA) combined as a single population. Subjects are controls (black, n=20), simplex PLA (blue, n=16), and systemic PLA (red, n=12). No differences between patient groups were found except that CD4-CCR10, CD8-CCR10 and CD8-CCR4 expression were suppressed in simplex patients (P=0.02, 0.04 and P<0.001 by two-way ANOVA).

Figure S5. Albumin and immunoglobulin levels in PLA subjects

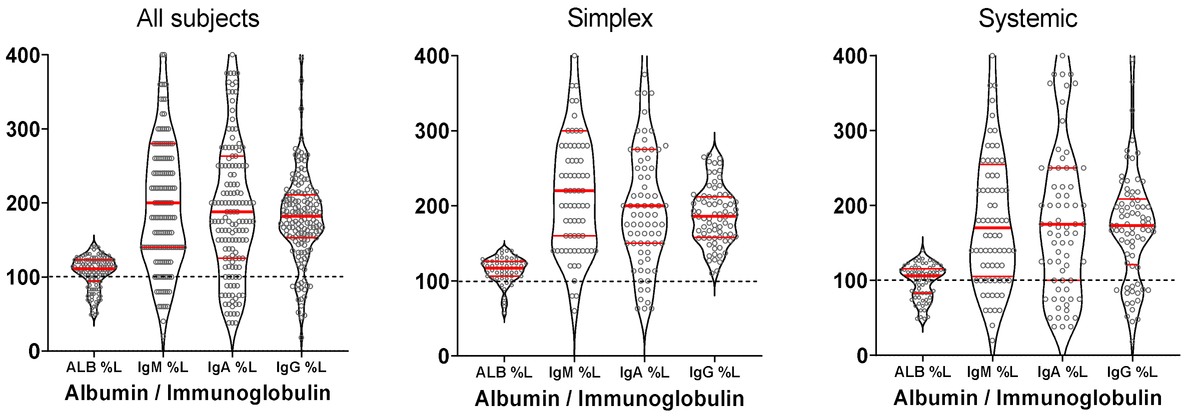

Violin plots show individual values expressed relative to the lower limit of normal (as percentage, %L); red horizontal lines represent medians and quartiles; dashed line represents the lower limit of normal. IgM, immunoglobulin M; IgA, immunoglobulin A; IgG, immunoglobulin G. Data are from the retrospective cohort; n=177 of whom 155 had data for immunoglobulin and 129 had data for albumin levels.
